# Effects of Visual Feedback Availability on Aerobic Performance and Pacing Strategy During a 5 km Running Time Trial

**DOI:** 10.3390/sports14070278

**Published:** 2026-07-03

**Authors:** Lucas Henrique Gonçalves de Brito, Anderson Geremias Macedo, Autran José da Silva Júnior, Tiago André Freire de Almeida, Danilo Alexandre Massini, Dalton Muller Pessôa Filho, Wonder Passoni Higino

**Affiliations:** 1 Postgraduate Program in Rehabilitation Sciences, Institute of Motricity Sciences, Federal University of Alfenas (UNIFAL), Santa Clara Campus, Avenida Jovino Fernandes Sales, Alfenas 37130-001, MG, Brazil; lucasbritogoncalv@gmail.com (L.H.G.d.B.); andersongmacedo@yahoo.com.br (A.G.M.); wonder.higino@unifal-mg.edu.br (W.P.H.); 2 University Center of the Guaxupé Educational Foundation (UNIFEG), Av. Dona Floriana, Guaxupé 37800-000, MG, Brazil; autranjsilvajr@gmail.com; 3 Centre for Research in Economics and Comparative Development (CIEDEC), Lusíada University of Lisbon, 1349-001 Lisboa, Portugal; tiagofalmeida@lis.ulusiada.pt; 4 Department of Physical Education, College of Sciences (FC), São Paulo State University (UNESP), Bauru 17033-360, SP, Brazil; dmassini@hotmail.com; 5 Post-Graduate Program in Human Development and Technology, Institute of Biosciences, São Paulo State University (UNESP), Rio Claro 13506-900, SP, Brazil; 6 Research and Study Group in Health Sciences (GEP-CS) of the Federal Institute of Education (IB), Science and Technology of Southern Minas Gerais (IFSULDEMINAS), Muzambinho Campus, Morro Preto 37890000, MG, Brazil

**Keywords:** physiological responses, fixed distance, 5-km run, pacing strategy

## Abstract

Running is a widely practiced exercise modality in which central and peripheral fatigue can influence performance and pacing strategy. This study investigated the influence of cognitive–emotional factors, based on the psychobiological model of fatigue, on 5 km time trial performance using a randomized crossover design. Twenty-two recreational male runners (23.0 ± 3.05 years) completed four laboratory visits. During the first visit, participants underwent body composition assessment and an incremental test to determine maximal oxygen uptake (VO_2_max) and the velocity associated with VO_2_max. In the subsequent three visits, participants performed a 5 km treadmill time trial as fast as possible under three conditions: no feedback (5k-NF), distance-only feedback (5k-Dist), and full feedback (5k-FF). No significant differences in performance were observed between conditions (5k-FF: 24.3 ± 1.8 min; 5k-NF: 24.8 ± 2.1 min; 5k-Dist: 24.7 ± 2.7 min). Regardless of the condition, ratings of perceived exertion and heart rate increased progressively throughout the trials. Other physiological variables showed similar responses across conditions. These findings indicate that manipulating feedback availability during a 5 km time trial did not significantly alter performance or physiological responses under the specific laboratory conditions examined, despite that the true absolute absence of effect should be interpreted with appropriate caution.

## 1. Introduction

Completing a running task in the shortest possible time requires appropriate regulation of exercise intensity throughout the event. This regulation, known as pacing strategy, aims to optimize performance by preserving energy reserves and preventing the premature onset of fatigue [1]. In this context, neuromuscular fatigue is defined as the inability to maintain a given level of force and may be influenced by neural and/or muscular factors [2]. Traditionally, fatigue has been classified as central or peripheral, depending on the volume, intensity, nature, and type of exercise. Peripheral fatigue is primarily associated with short-duration, high-intensity exercise, whereas central fatigue is more commonly observed during prolonged exercise of relatively lower intensity. Central fatigue involves alterations in neural drive to the skeletal muscle, while peripheral fatigue encompasses mechanisms distal to the neuromuscular junction [2,3].

Several theoretical models have been proposed to explain the mechanisms underlying fatigue. The catastrophic model, introduced by Archibald Hill in 1923, suggests that limitations in oxygen supply to the muscle lead to a progressive decline in work capacity until exercise cessation [4]. In contrast, the central governor model, proposed by Tim Noakes [5], posits that the central nervous system anticipatorily regulates effort to prevent physiological failure by dynamically adjusting the exercise intensity. In this framework, ratings of perceived exertion (RPE) play a key role in exercise regulation, enabling individuals to monitor effort intensity and decide whether to continue or terminate the task [6].

Building on these perspectives, the psychobiological model of fatigue, proposed by Samuele Marcora and further discussed by Pageaux et al. [7], integrates physiological and cognitive-emotional factors in the determination of performance. This model highlights five main components: (i) RPE, (ii) potential motivation, (iii) knowledge of task duration or distance, (iv) knowledge of task progress, and (v) prior experience. In time-to-exhaustion tasks, the decision to stop is primarily influenced by the interaction between RPE and potential motivation. In contrast, in tasks with a predetermined endpoint, such as time trials, pacing regulation involves more complex decision-making processes, including anticipation and continuous adjustments in intensity based on available feedback and prior experience [8].

Accordingly, pacing strategy is closely linked to the psychobiological model of fatigue [9]. In closed-loop tasks, in which the endpoint is known in advance, the central nervous system uses this feedback to regulate effort throughout the exercise bout, acting as a control system that integrates physiological and cognitive signals [10,11,12]. This perspective reinforces the notion that physical performance is not determined solely by metabolic limitations, but also by perceptual, motivational, and decision-making processes.

Although previous feedback manipulation studies have advanced our understanding of pacing, empirical evidence regarding the role of sensory feedback in running remains inconsistent and heavily reliant on cycling protocols or elite populations. For instance, while some cycling models suggest a tight dependence on external cues, visual deprivation and auditory manipulations during longer trials have shown that pacing regulation can occur relatively independent of external feedback [13]. However, a significant gap remains regarding how different levels of feedback availability influence effort regulation during moderate-duration running. Investigating a 5 km treadmill time trial in recreationally trained runners offers a unique and novel contribution because: (1) running involves distinct, high-impact dynamic sensory inputs compared to cycling; (2) the 5 km distance represents one of the most commercially popular and widely practiced running modalities, maximizing the ecological relevance of the findings; and (3) recreational runners may possess distinct cognitive-pacing behaviors and varying levels of internal programming compared to elite athletes. Therefore, understanding these responses under simultaneous psychophysiological monitoring is crucial to clarifying the boundaries of the psychobiological model of fatigue in everyday athletic contexts [14,15,16,17].

Therefore, the present study aimed to investigate the influence of psychobiological factors of fatigue on performance during a 5 km running time trial. Specifically, pacing strategy and physiological responses were examined under conditions with different levels of feedback. It was hypothesized that restricting feedback would impair performance and alter the pacing strategy. Additionally, it was hypothesized that RPE would be higher under reduced feedback conditions due to increased perceptual uncertainty during exercise.

## 2. Materials and Methods

### 2.1. Participants

The present study was designed as a randomized crossover trial. Participants were recruited through advertisements in running clubs, fitness centers, institutional boards, and social media platforms (Facebook, Instagram, and WhatsApp running groups) during the data collection period.

Twenty-two non-elite male runners regularly engaged in running training volunteered to participate in this study. To better characterize their training status, participants reported an average weekly running mileage of 20 ± 5.3 km·week^−1^, a training history of 1.5 ± 0.5 years in road running, and an average personal best time for a 5 km distance of 24:19 ± 2:43 min. All participants met the following inclusion criteria: (a) age between 20 and 30 years; (b) regular practice of road running for at least 6 months; (c) prior participation in at least one event of ≥5 km; (d) minimum training frequency of three sessions per week; (e) absence of clinical conditions or injuries that could impair test performance; (f) no use of supplements or medications with potential stimulatory effects on the central nervous system; and (g) provision of written informed consent. Exclusion criteria included: (a) failure to attend or complete any stage of the study; (b) occurrence of injury or health conditions preventing continuation; and (c) voluntary withdrawal at any time.

All procedures were approved by the institutional Research Ethics Committee (CAAE: 67726123.9.0000.5142) and conducted in accordance with ethical standards for research involving human participants.

### 2.2. Experimental Design

**Figure 1:**
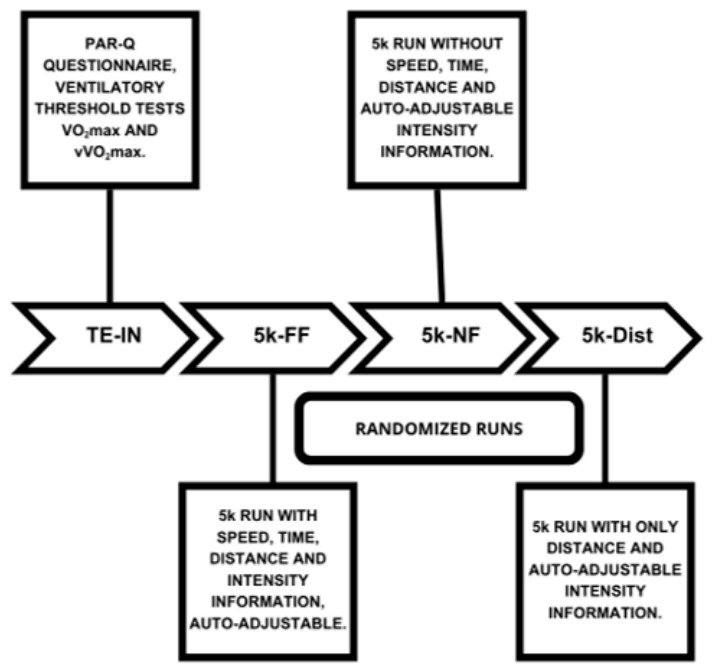
Experimental design of the study. TE-IN: incremental test; 5k-FF: 5 km time trial with full feedback; 5k-NF: 5 km time trial with no feedback; 5k-Dist: 5 km time trial with distance-only feedback.

Each participant completed four laboratory visits separated by 24–72 h (Figure 1). During the first visit, participants completed the Physical Activity Readiness Questionnaire (PAR-Q), followed by body composition assessment and an incremental treadmill test to determine maximal oxygen uptake (VO_2max_) and the associated velocity (vVO_2max_).

To control information availability during the 5 km time trials, the treadmill console was physically and completely covered using opaque sheets, ensuring that participants could not visually perceive any performance metrics unless dictated by the experimental condition. In the Blind condition (5k-SI), all visual feedback (speed, elapsed time, and covered distance) was completely hidden. In the Distance-only condition (5k-Dist), only the distance covered was made visible through a customized opening in the cover. In the Full-feedback condition (5k-CI), the console remained fully uncovered, displaying speed, time, and distance. To prevent incomplete blinding and ensure strict experimental separation, no verbal cues, feedback, or time-splits were provided by the researchers during any of the trials. Additionally, while participants could naturally perceive the general mechanical sound and belt acceleration inherent to motorized treadmills, they were blinded to the exact digital speed increments, relying solely on their subjective perception of effort to freely adjust the pace. In all conditions, participants were strongly instructed to complete the 5 km distance as fast as possible.

The order of the three experimental conditions was randomized and fully counterbalanced among participants. Prior to the trials, all six possible experimental sequences involving the three conditions (5k-NF, 5k-Dist, and 5k-FF) were generated, and participants were randomly allocated to one of these sequences using a simple randomization procedure. Consequently, the 22 participants were distributed across the six possible counterbalanced condition sequences as follows: Sequence 1 (5k-NF → 5k-Dist → 5k-CI), n = 4; Sequence 2 (5k-NF → 5k-FF → 5k-Dist), n = 5; Sequence 3 (5k-Dist → 5k-NF → 5k-FF), n = 4; Sequence 4 (5k-Dist → 5k-FF → 5k-FF), n = 3; Sequence 5 (5k-FF → 5k-NF → 5k-Dist), n = 3; and Sequence 6 (5k-FF → 5k-Dist → 5k-NF), n = 3. Therefore, a near-balanced counterbalancing was achieved to mitigate potential order, learning, or adaptation effects. Due to this strict counterbalancing design, order effects were structurally neutralized, and sequence was not included as a separate statistical factor.

No separate familiarization trials were performed before the testing protocol. Although formal familiarization sessions were not structured prior to the experimental trials, potential learning or novelty effects were systematically controlled. This was achieved by strictly recruiting recreationally trained runners with extensive, documented experience in treadmill running. Additionally, to ensure protocol consistency, participants received a standardized, comprehensive briefing before each testing session, which detailed the warm-up procedures, the metabolic gas-analyzer interfaces, and the treadmill’s manual speed-adjustment mechanics.

During all experimental tests, the environmental conditions in the laboratory were kept constant (temperature: ~22 °C; relative humidity: ~50%), and all participants were required to perform all trials at the same period, as well as were instructed to maintain their typical sleep patterns, avoid strenuous physical exercise for 24 h prior to testing, and abstain from caffeine, alcohol, and nutritional supplements for at least 12 h before each experimental session.

### 2.3. Body Composition

Body composition was assessed using a multifrequency octapolar bioelectrical impedance analyzer (InBody 720, Biospace Co., Ltd., Seoul, Republic of Korea), providing measurements of skeletal muscle mass (SMM), body fat percentage (%BF), fat mass (FM), and total body mass (TBM). Participants followed standardized pre-assessment guidelines as described by Parra et al. [18]. Height (h) was measured using a portable stadiometer (Standard, Sanny, São Paulo, Brazil) according to established procedures [19].

### 2.4. Incremental Test

The incremental treadmill test was performed to voluntary exhaustion and preceded by a standardized warm-up followed by rest. The initial speed was set between 7 and 9 km·h^−1^ according to individual fitness level, with increments of 1 km·h^−1^ every 2 min. Treadmill incline was maintained at 1% to simulate outdoor running conditions.

Ventilatory variables were continuously measured using a gas exchange analyzer (VO2000, Aerosport, Medgraphics, St. Paul, MN, USA), including oxygen uptake (VO_2_), carbon dioxide production (VCO_2_), pulmonary ventilation (VE), and respiratory exchange ratio (RER). Heart rate (HR) and ratings of perceived exertion (RPE) were recorded at the end of each stage.

VO_2max_ was defined as the highest 30 s average VO_2_ obtained during the final stages of the test. The following criteria were used: (i) VO_2_ plateau (increase <150 mL·min^−1^ despite increased workload); (ii) RER ≥ 1.10; and (iii) HR within 10 bpm of age-predicted maximum (220age). When these criteria were not met, the highest VO_2_ value was considered VO_2_peak [20].

The velocity associated with VO_2max_ (vVO_2max_) was defined as the lowest speed at which VO_2max_ was achieved. When the final stage was not completed, vVO_2max_ was calculated according to the equation proposed by Kuipers et al. [21]:

vVO_2max_ = *v* + (*t*/120) × *I*

where *v* is the speed of the last completed stage, *t* is the time (s) sustained in the uncompleted stage, and *I* is the speed increment (1 km·h^−1^).

### 2.5. Standardized Warm-Up

Before all experimental procedures (incremental test and 5 km trials), participants performed a standardized treadmill warm-up consisting of 10 min of light-intensity running, corresponding to values between 9 (“very light”) and 11 (“light”) on the Borg RPE scale.

### 2.6. 5 km Time Trial

Following the warm-up, participants were instructed to complete a 5 km run in the shortest possible time. The test was performed on a motorized treadmill, and participants were allowed to self-regulate exercise intensity by adjusting speed according to perceived effort and pacing strategy.

Heart rate (HR), ratings of perceived exertion (RPE), and split time were recorded at each kilometer and used for pacing analysis.

As previously described, participants completed three 5 km time trials under different conditions: (i) no feedback (5k-NF), (ii) distance-only feedback (5k-Dist), and (iii) full feedback (5k-FF).

During all trials, participants were equipped with a gas exchange system (VO2000, Aerosport, Medgraphics, St. Paul, MN, USA), enabling continuous measurement of ventilatory variables (VO_2_, VCO_2_, VE, and RER), as in the incremental test.

### 2.7. Rating of Perceived Exertion

RPE was assessed at the end of each stage of the incremental test and at each kilometer during the 5 km trials using the Borg 6–20 scale [22], adapted for the Brazilian context [23].

### 2.8. Statistical Analysis

All variables are presented as the mean ± standard deviation. Data normality (Shapiro–Wilk test), homogeneity of variances (Levene test), and sphericity (Mauchly’s test) were assessed.

Given that assumptions were met, a two-way repeated-measures ANOVA (condition × distance) with Tukey’s post hoc test was used to examine the effects of the three running conditions (5k-FF, 5k-Dist, and 5k-NF) across time points (1st to 5th km).

To compare the total time to complete the 5 km trial between conditions, a one-way ANOVA with Tukey’s post hoc test was performed.

The post-hoc statistical power analysis was computed using G*Power software (version 3.1.9.7) to mathematically justify the adequacy of our sample size. The analysis for a two-way repeated-measures ANOVA (three feedback conditions × five sampling moments), involved the final sample of 22 recreationally trained runners. Based on a conservative alpha level of α = 0.05 and a standard moderate effect size (f = 0.25), the post-hoc calculation yielded an actual statistical power of 0.83 (83%). Because this value comfortably exceeds the conventionally accepted scientific threshold of 0.80, we can confidently assert that the sample size was statistically sufficient to detect meaningful differences between the feedback conditions if they existed. Consequently, the observed null findings represent a true lack of experimental effect rather than a limitation in statistical power, minimizing the probability of Type II error.

All analyses were conducted using JAMOVI software (version 2.3.21), with statistical significance set at *p* ≤ 0.05.

## 3. Results

Table 1 presents the descriptive characteristics of the sample (mean ± standard deviation).

**Figure 2:**
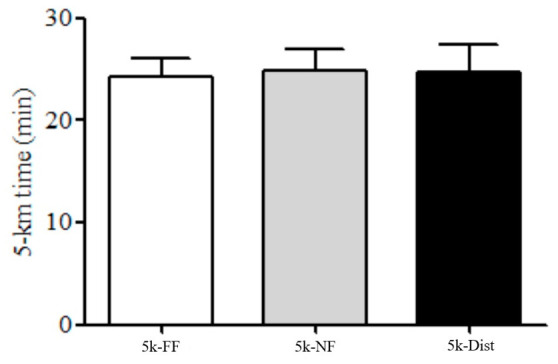
Total time to complete the 5 km time trial across conditions (5k-FF, 5k-NF, and 5k-Dist). Values are presented as mean ± SD. *p* > 0.05.

**Figure 3:**
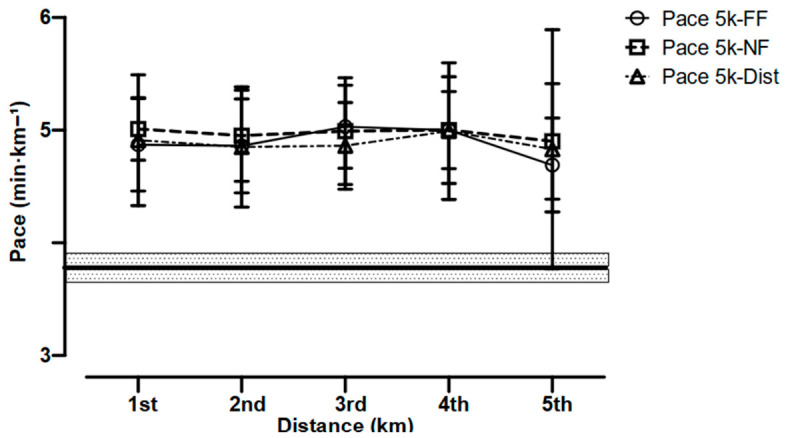
Pacing (min·km^−1^) from the 1st to the 5th kilometer during the 5 km time trial across conditions (5k-FF, 5k-NF, and 5k-Dist; n = 22). Values are presented as mean ± SD. The solid horizontal line and shaded area represent the mean ± SD pacing corresponding to the velocity associated with VO_2max_ (vVO_2max_). *p* > 0.05.

**Figure 4:**
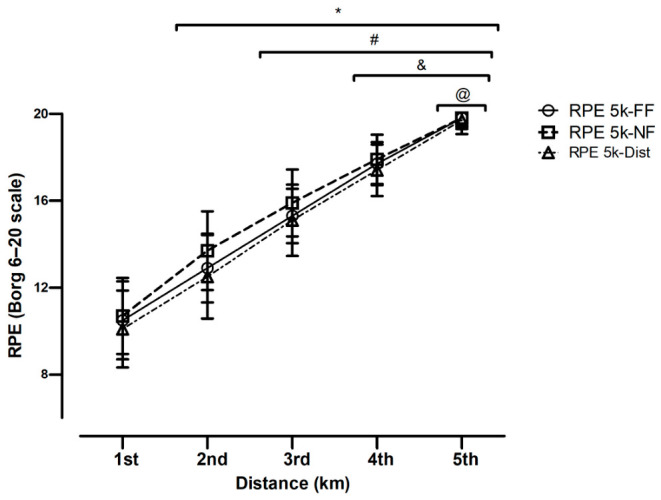
Ratings of perceived exertion RPE (Borg 6–20 scale) from the 1st to the 5th kilometer during the 5 km time trial across conditions (5k-FF, 5k-NF, and 5k-Dist; n = 22). Values are presented as the mean ± SD. * Significantly different from the 1st kilometer; # significantly different from the 2nd kilometer; & significantly different from the 3rd kilometer; @ significantly different from the 4th kilometer (within each condition; *p* < 0.05).

**Figure 5:**
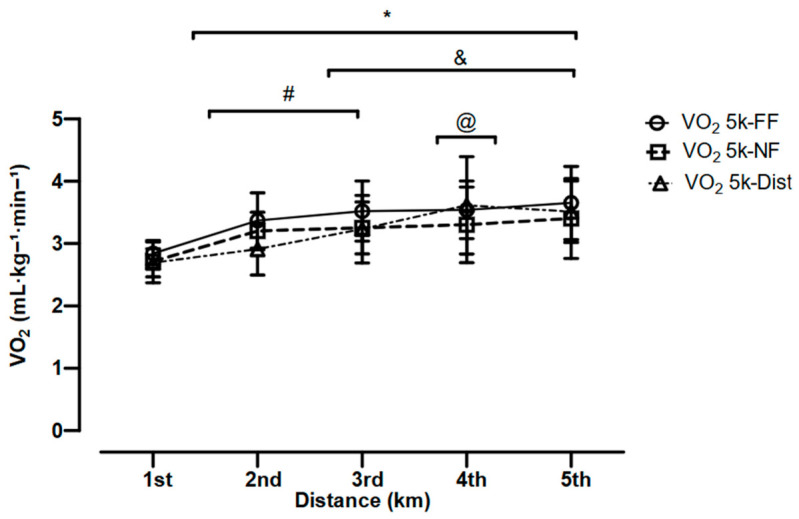
Oxygen uptake (VO_2_, mL·kg^−1^·min^−1^) across the 5 km time trial under different conditions (5k-FF, 5k-NF, and 5k-Dist; n = 22). Values are presented as the mean ± SD. * Significantly different from the 1st kilometer in 5k-FF; # significantly different from the 1st kilometer in 5k-NF; & significantly different from the 1st kilometer in 5k-Dist; @ significantly different from the 3rd kilometer in 5k-Dist (*p* < 0.05).

**Figure 6:**
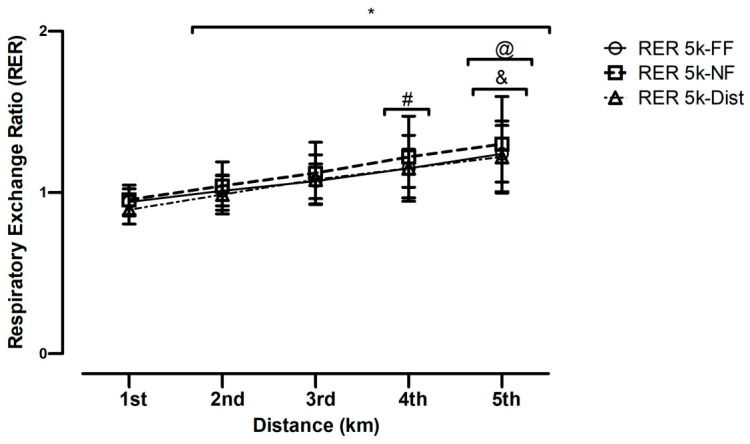
Respiratory exchange ratio (RER) across the 5 km time trial under different conditions (5k-FF, 5k-NF, and 5k-Dist; n = 22). Values are presented as the mean ± SD. * Significantly different from the 1st kilometer in all conditions; # significantly different from the 2nd kilometer in 5k-NF and 5k-Dist; & significantly different from the 3rd kilometer in 5k-NF; @ significantly different from the 4th kilometer in all conditions (*p* < 0.05).

**Figure 7:**
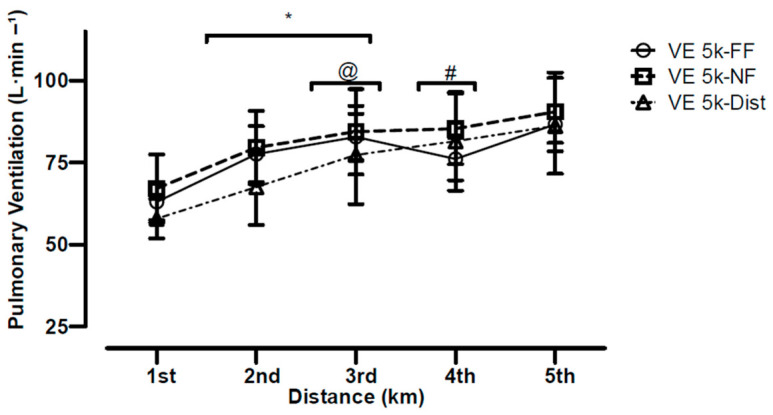
Pulmonary ventilation (VE, L·min^−1^) across the 5 km time trial under different conditions (5k-FF, 5k-NF, and 5k-Dist; n = 22). Values are presented as the mean ± SD. * Significantly different from the 1st kilometer in all conditions; # significantly different from the 1st kilometer in 5k-NF and 5k-Dist; @ significantly different from the 2nd kilometer in all conditions (*p* < 0.05).

**Figure 8:**
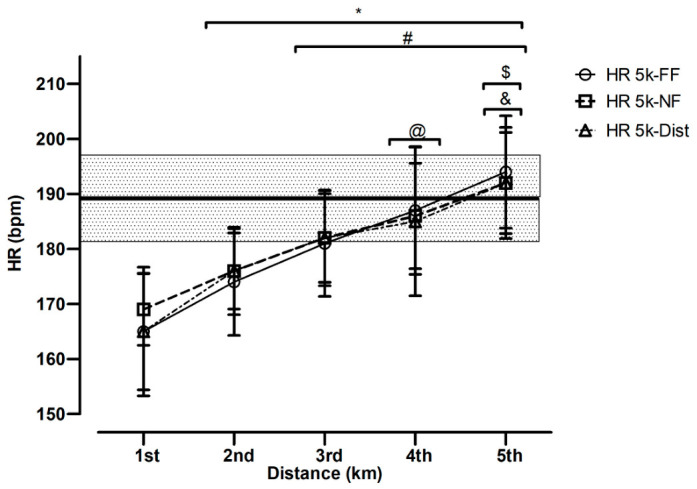
Heart rate (HR, bpm) across the 5 km time trial under different conditions (5k-FF, 5k-NF, and 5k-Dist; n = 22). Values are presented as the mean ± SD. The solid horizontal line and shaded area represent the mean ± SD HR corresponding to the maximal heart rate (HRmax). * Significantly different from the 1st kilometer in all conditions; # significantly different from the 2nd kilometer in all conditions; @ significantly different from the 3rd kilometer in 5k-FF; $ significantly different from the 3rd kilometer in all conditions; & significantly different from the 4th kilometer in all conditions (*p* < 0.05).

Figure 2, Figure 3, Figure 4, Figure 5, Figure 6, Figure 7 and Figure 8 present the variables analyzed during the 5 km treadmill time trial under the three conditions: 5k-FF, 5k-NF, and 5k-Dist. Figure 2 shows the total time to complete the 5 km time trial (mean ± SD) across conditions. No significant differences were observed between conditions.

Figure 3 shows pacing (min·km^−1^) during the 5 km time trial across conditions (5k-FF, 5k-NF, and 5k-Dist). No significant differences were observed across distances within each condition or between the experimental conditions (*p* > 0.05). The repeated-measures ANOVA revealed a significant main effect of time on running pace [F(3.44, 134.33) = 3.70, *p* = 0.010, η_p_^2^ = 0.032]. However, no significant main effect was found for the experimental condition [F(2, 39) = 0.20, *p* = 0.822, = 0.01], nor was there a significant condition × time interaction [F(6.89, 134.33) = 0.58, *p* = 0.767, η_p_^2^ = 0.010]. Consequently, these findings demonstrate the absence of a condition × distance interaction, indicating that the pacing profiles remained uniform across all experimental sessions and that variations in running pace over the 5 km trial occurred independently of feedback availability.

Despite that no significant differences were observed for ratings of perceived exertion (RPE) (Figure 4) among the evaluated conditions, the values of RPE increased progressively and significantly across distances in all conditions (*p* < 0.05). The repeated-measures ANOVA revealed a significant main effect of time [F(2.55, 99.61) = 802.44, *p* < 0.001, η_p_^2^ = 0.887]. Conversely, no significant main effect was found for the experimental condition [F(2, 39) = 2.10, *p* = 0.137, η_p_^2^ = 0.062], nor was there a significant condition × time interaction [F(5.11, 99.61) = 0.91, *p* = 0.483, η_p_^2^ = 0.017].

Figure 5 shows VO_2_ (mL·kg^−1^·min^−1^) during the 5 km time trial across conditions (5k-FF, 5k-NF, and 5k-Dist). The repeated-measures ANOVA revealed a significant main effect of time [F(1.69, 65.95) = 47.36, *p* < 0.001, η_p_^2^ = 0.253], indicating alterations in the variable across the evaluated periods. Conversely, no significant main effect was observed for the experimental condition [F(2, 39) = 0.39, *p* = 0.681, η_p_^2^ = 0.014]. Additionally, there was no significant condition × time interaction [F(3.38, 65.95) = 1.34, *p* = 0.266, η_p_^2^ = 0.019]. However, significant differences were found across distances within each condition (*p* < 0.05). In the 5k-FF condition, VO_2_ values at the 2nd, 3rd, 4th, and 5th kilometers were significantly higher than at the 1st kilometer. In the 5k-NF condition, VO_2_ at the 2nd and 3rd kilometers was significantly higher than at the 1st kilometer. In the 5k-Dist condition, VO_2_ at the 3rd, 4th, and 5th kilometers was significantly higher than at the 1st kilometer, and the 4th kilometer was also significantly higher than at the 3rd kilometer.

Figure 6 shows the respiratory exchange ratio (RER) during the 5 km time trial across conditions (5k-FF, 5k-NF, and 5k-Dist). The repeated-measures ANOVA revealed a significant main effect of time [F(1.50, 58.34) = 81.67, *p* < 0.001, η_p_^2^ = 0.321], indicating alterations in the variable across the evaluated periods. Conversely, no significant main effect was observed for the experimental condition [F(2, 39) = 0.77, *p* = 0.471, η_p_^2^ = 0.030]. Similarly, there was no significant condition × time interaction [F(2.99, 58.34) = 0.39, *p* = 0.761, η_p_^2^ = 0.004]. However, significant differences were found across distances within each condition (*p* < 0.05). In all conditions (5k-FF, 5k-NF, and 5k-Dist), RER values from the 2nd to the 5th kilometer were significantly higher than at the 1st kilometer. Additionally, in the 5k-NF and 5k-Dist conditions, the 4th kilometer was significantly higher than the 2nd kilometer. In the 5k-NF condition, the 5th kilometer was significantly higher than the 3rd kilometer. Finally, the 5th kilometer was significantly higher than the 4th kilometer in all conditions.

Figure 7 shows pulmonary ventilation (VE, L·min^−1^) during the 5 km time trial across conditions (5k-FF, 5k-NF, and 5k-Dist). The repeated-measures ANOVA revealed a significant main effect of time [F(2.06, 80.35) = 130.43, *p* < 0.001, η_p_^2^ = 0.419], indicating alterations in the variable across the evaluated periods. No significant main effect was observed for the experimental condition [F(2, 39) = 2.05, *p* = 0.142, η_p_^2^ = 0.076]. Furthermore, the condition × time interaction did not reach statistical significance [F(4.12, 80.35) = 2.45, *p* = 0.051, η_p_^2^ = 0.026]. However, significant differences were found across distances within each condition (*p* < 0.05). In all conditions, VE at the 2nd and 3rd kilometers was significantly higher than at the 1st kilometer. Additionally, in the 5k-NF and 5k-Dist conditions, the 4th kilometer was significantly higher than the 1st kilometer. Furthermore, in all conditions, the 3rd kilometer was significantly higher than the 2nd kilometer.

Figure 8 shows the heart rate (HR, bpm) during the 5 km time trial across conditions (5k-FF, 5k-NF, and 5k-Dist). The repeated-measures ANOVA revealed a significant main effect of time [F(2.34, 91.19) = 102.83, *p* < 0.001, η_p_^2^ = 0.384]. Conversely, no significant main effect was observed for the experimental condition [F(2, 39) = 0.29, *p* = 0.749, η_p_^2^ = 0.011] nor was there a significant condition × time interaction [F(4.68, 91.19) = 1.18, *p* = 0.323, η_p_^2^ = 0.014]. However, significant differences were found across distances within each condition (*p* < 0.05). In all conditions, HR from the 2nd to the 5th kilometer was significantly higher than at the 1st kilometer. Similarly, HR from the 3rd to the 5th kilometer was significantly higher than at the 2nd kilometer. Additionally, the 4th kilometer in the 5k-FF condition and the 5th kilometer in all conditions were significantly higher than the 3rd kilometer. Finally, the 5th kilometer was significantly higher than the 4th kilometer in all conditions.

## 4. Discussion

The present study investigated the influence of psychobiological factors of fatigue on performance, pacing strategy, and physiological responses during a 5 km running time trial. The main findings indicate that manipulation of external feedback (distance, time, and speed) did not exert a significant statistical effect on performance, pacing, ratings of perceived exertion (RPE), or physiological responses throughout the exercise, despite that a high power value for moderate effects does not mathematically equate to proof of a true zero effect, as smaller effects could indeed remain undetected within our sample size.

These findings are consistent with previous studies examining the role of sensory feedback in pacing regulation. In cyclists, the absence of visual stimuli and manipulation of auditory cues did not alter performance, heart rate, or RPE during a 40 km time trial, suggesting that effort regulation is predominantly supported by internal factors, such as perceived exertion and prior knowledge of the task [13]. Similarly, the presence of another runner did not affect performance, heart rate, or RPE during a 5 km run, despite a lower perceived difficulty reported by participants [24]. Together, these findings suggest that pacing strategy is relatively robust to external manipulations. In this context, prior knowledge of the task and experience with similar efforts appear to play a central role in pacing regulation. Evidence indicates that individuals rely on an internal clock to estimate elapsed time and adjust the exercise intensity, even in the absence of direct external feedback [13]. In the present study, the maintenance of performance across conditions may be explained by participants’ familiarity with the 5 km distance and their ability to use internal cues, such as RPE, to regulate effort. However, this interpretation should be considered in light of the sample characteristics and task duration, limiting broader generalization [25].

Similar results have been reported in studies manipulating feedback accuracy. Albertus et al. [26] showed that trained cyclists did not alter performance despite inaccurate distance feedback, suggesting the adoption of a pre-established pacing strategy. In the present study, although performance was unchanged, RPE increased progressively throughout the trial. These findings are consistent with the psychobiological model of fatigue, in which performance is centrally regulated through the interaction between perceived effort, motivation, and prior experience.

Furthermore, the lack of significant differences among conditions should be interpreted with caution and under the framework of the study’s boundaries. First, recreational runners might rely more predominantly on raw internal perceived exertion (RPE) and distinct cognitive-pacing behaviors compared to elite athletes, who are typically trained to utilize precise external cues to partition energy over split times [1,27]. Second, the 5 km distance duration (~24 min in our sample) might not be sufficiently prolonged to fully amplify or accumulate the psychobiological effects of feedback deprivation or uncertainty [12,27]. Finally, the laboratory setting itself restricts real-world environmental variability, potentially flattening behavioral and physiological responses that would typically occur outdoors [28].

However, not all studies report lack of feedback effects. Mauger et al. [27] observed improved performance in cyclists when real-time feedback was provided, possibly due to increased motivation and modulation of perceived exertion. In contrast, the present results suggest that, in self-paced 5 km running, external feedback may have limited influence on effort regulation, despite progressive increases in RPE and heart rate.

Additionally, evidence suggests that cognitive factors can influence performance without necessarily altering physiological responses. Studies have shown that mental fatigue can impair performance without significantly changing variables such as heart rate [9,29]. In the present study, although heart rate increased progressively, performance remained stable across conditions, indicating that participants regulated effort primarily based on perceptual cues and prior experience. These findings reinforce the role of cognitive and perceptual processes, such as task knowledge and anticipatory regulation, in pacing.

From this perspective, the psychobiological model of fatigue provides a consistent framework for interpreting the present findings. This model proposes that endurance performance is regulated by the interaction between perceived exertion and motivation, mediated by cognitive control processes [30,31]. Brain regions such as the prefrontal cortex and anterior cingulate cortex are involved in decision-making, fatigue monitoring, and adjustment of exercise intensity [29,32,33]. The progressive increase in RPE across all conditions, regardless of feedback availability, supports the notion that performance regulation was predominantly driven by internal mechanisms.

Overall, the present findings indicate that endurance performance results from the interaction between physiological and psychobiological factors. The absence of feedback effects suggests that effort regulation was primarily guided by internal signals, particularly RPE, motivation, and prior experience, which appear to sustain pacing even in the absence of external feedback. Furthermore, the progressive increase in RPE, independent of substantial differences in cardiorespiratory variables, reinforces its central role in exercise regulation [7,34].

Therefore, the current findings can be robustly interpreted through the anticipatory regulation and teleoanticipation theories within contemporary cognitive regulation models of endurance performance. According to predictive processing perspectives, the brain does not merely respond passively to incoming sensory or external feedback; instead, it generates proactive, top-down predictions regarding the metabolic costs of the task based on prior experience and internal homeostatic status [4,29,30]. In the current study, the preservation of pacing profiles and physiological responses—even during total feedback deprivation—suggests that recreational runners relied fundamentally on these pre-determined internal templates. The internal teleoanticipatory clock and the dynamic monitoring of ratings of perceived exertion (RPE) appeared sufficient to safely regulate exercise intensity throughout the 5 km distance, effectively diminishing the practical reliance on real-time external digital cues, as previously theorized in comprehensive frameworks of biological information processing during exercise [35].

This study has some limitations that should be acknowledged. First, the sample consisted exclusively of male recreational runners, which may limit the generalizability of the findings to female participants or highly trained athletes. Second, the experimental protocol was conducted on a motorized treadmill, which lacks the environmental ecology of overground running. A previous study has highlighted that although mathematical threshold parameters (such as critical velocity) can yield statistically identical values between track and treadmill conditions, track-based assessments exhibit superior specificity for athletic performance control and training prescription [28]. Running on a motorized belt eliminates the mechanical requirement of accelerating one’s own body mass against air resistance and significantly reduces speed fluctuations and environmental variability [36]. These mechanical discrepancies alter the energy cost of running and the dynamic integration of cardiorespiratory responses, which may consequently modify individual physiological and perceptual coupling compared to real-world time trials [28]. Therefore, caution should be exercised when extrapolating treadmill-derived null effects to outdoor environments [37]. Third, the protocol did not include a formal laboratory familiarization session prior to the experimental trials. Although all participants were highly experienced road runners accustomed to treadmill training and received strictly standardized instructions, a dedicated familiarization trial might have further reduced any residual learning effects related to treadmill speed adjustment or the experimental procedures. Nevertheless, the randomized crossover design, together with the participants’ previous experience, likely minimized the influence of such effects across conditions. Lastly, these findings are highly specific to the sample profile evaluated in this study—recreational male runners. Consequently, these results should not be indiscriminately generalized to female runners, elite athletes, or individuals with distinct training statuses and competitive experience, as pacing strategy and psychological resilience to feedback manipulation can vary substantially across different demographics and fitness levels. In addition, it is important to acknowledge that while the post-hoc power analysis indicated adequate strength to detect moderate-to-large effects, it does not definitively confirm a true, absolute absence of effect.

Furthermore, it is important to note that although the present findings are discussed in light of the psychobiological model of fatigue, the underlying cognitive-emotional mechanisms proposed by this model were not directly evaluated beyond ratings of perceived exertion (RPE). Variables such as potential motivation, prior experience, mental fatigue, affective valence, attentional focus, perceived task difficulty, and cognitive control were not measured in the current study. Therefore, any reference to these constructs should be interpreted as theoretical inferences rather than direct assessments, which somewhat limits a deeper interpretation of the cognitive-emotional component emphasized in our introduction. Consequently, future studies should look beyond external feedback and directly quantify cognitive control demands (e.g., mental fatigue, executive function) alongside psychological traits as primary determinants of competitive pacing strategies. Crucially, future designs should evaluate how these behavioral and cognitive modifications dynamically dictate aerobic performance and physiological dependency (such as metabolic rate and oxygen kinetics), thereby elucidating the direct impact of cognitive regulation over physiological outcomes in endurance running.

## 5. Conclusions

In conclusion, manipulating external feedback availability (distance or full information) did not significantly alter 5 km running time trial performance, pacing strategy, rating of perceived exertion, or physiological responses under the conditions examined in recreationally trained runners. However, some physiological and perceptual responses observed, as heart rate and ratings of perceived exertion increased progressively and identically across all conditions. These findings suggest that when athletes are well-habituated to the exercise modality, pacing strategies and effort regulation are effectively driven by the interaction between physiological and psychobiological components, independent of real-time external feedback, despite smaller, subtle variations between conditions may have remained undetected, and thus, our non-significant findings should be interpreted with appropriate caution.

## Figures and Tables

**Table 1:** Descriptive characteristics of the participants (n = 22). Values are presented as the mean ± standard deviation (SD) and coefficient of variation (CV).

Variable	Mean ± SD	CV (%)
Age (years)	23.0 ± 3.05	13.2
Body Mass (kg)	73.7 ± 4.97	6.74
Height (cm)	177.00 ± 5.84	3.29
Body Fat (%)	11.3 ± 1.89	16.72
VO_2max_ (L·min^−1^)	4.26 ± 0.81	19.01
VO_2max_ (mL·kg^−1^·min^−1^)	56.1 ± 9.71	17.30
vVO_2max_ (km·h^−1^)	14.7 ± 1.05	7.14

## Data Availability

The data that support the findings of this study are available from the last author upon reasonable request.

## Abbreviations

The following abbreviations are used in this manuscript:

%G	Body Fat Percentage
HR	Heart Rate
RER	Respiratory Exchange Ratio
RPE	Perceived Exertion
TBM	Total Body Mass
VE	Pulmonary Ventilation
5k-Dist	5 km with Distance Feedback
5k-FF	5 km with Full Feedback
5k-NF	5 km No Feedback
